# Supplementary material for: Identification of photoperiod-induced specific miRNAs in the adrenal glands of Sunite sheep (Ovis aries)
Source: Front Vet Sci. 2022 Jul 22;9:888207. doi: 10.3389/fvets.2022.888207 (PMC9354845; doi:10.3389/fvets.2022.888207)
Supplement: Supplementary file 1 [file Data_Sheet_1.ZIP › Supplementary file/miRDeep2 file.zip!/miRDeep2 file/Novel_Structure/SPLP42A2/Novel_104.pdf]

Provisional ID	:	NC_019474.2_31479
Score total	:	1.5
Score for star read(s)	:	-1.3
Score for read counts	:	0
Score for mfe	:	1.2
Score for randfold	:	1.6
Score for cons. seed	:	
Total read count	:	279
Mature read count	:	279
Loop read count	:	0
Star read count	:	0

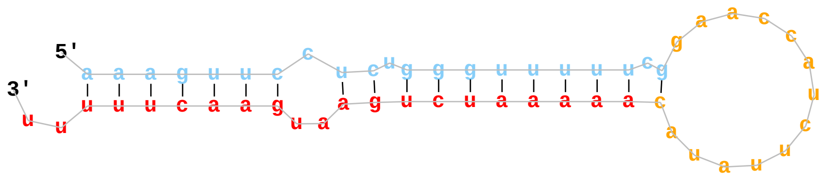

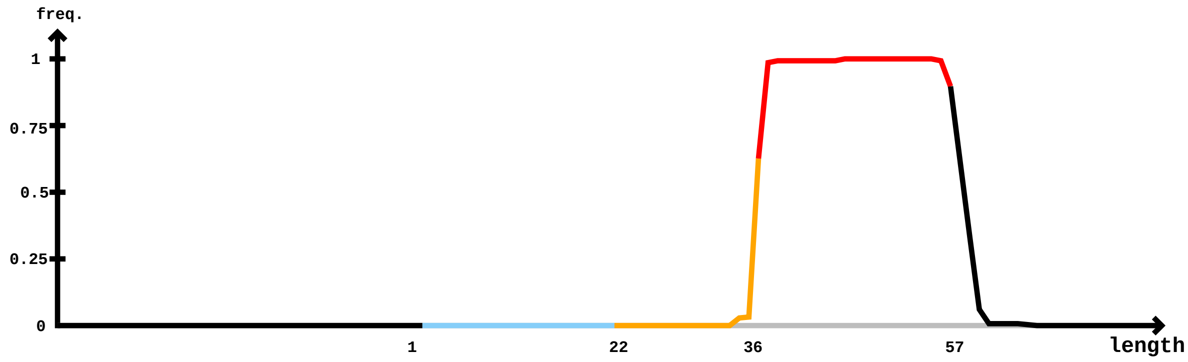

Star Mature

```
5'- gguuuauuacaggauauugaauauaugauuggccaaaaaguuccucuggguuuuucggaaccaucuuauacaaaaaucugaaugaacuuuuuggccaaccaguucccugugcu  -3'   exp
    .......((((((.....((((...((.(((((((((((((((.((.((((((((.(.............)))))))))))..))))))))))))))).))))))))))))..   reads   mm   sample
    .....................................................................aGaaaaaucugaaugaacuuuuu.....................   8       1    seq
    ......................................................................caaaaaucugaaugaacuuuuug....................   1       0    seq
    .......................................................................aaaaCucugaaugaacuuu.......................   2       1    seq
    .......................................................................aaaaaucugaaugaacuuuu......................   26      0    seq
    .......................................................................aaaaaucugaaugaGcuuuuu.....................   1       1    seq
    .......................................................................aaaaaucugaaugaacuuuuu.....................   76      0    seq
    .......................................................................aaaaaucugaaugaacCuuuu.....................   1       1    seq
    .......................................................................aaaaaucugaaugaacuuuuug....................   55      0    seq
    .......................................................................aaaaaucugaaugaacuuuuuU....................   4       1    seq
    .......................................................................aaaaaucugaaugaacuuuuuC....................   2       1    seq
    ........................................................................aaaCucugaaugaacuuuu......................   1       1    seq
    ........................................................................aaaaucuAaaugaacuuuuu.....................   2       1    seq
    ........................................................................aaaaucugaauAaacuuuuu.....................   1       1    seq
    ........................................................................aaaaucugaaugaacCuuuu.....................   1       1    seq
    ........................................................................aaaCucugaaugaacuuuuug....................   4       1    seq
    ........................................................................aaaaucugaaugaacuuuuuC....................   1       1    seq
    ........................................................................aaaaucugaaCgaacuuuuugg...................   2       1    seq
    ........................................................................aaaaucugaaugaacuuuuugA...................   66      1    seq
    ........................................................................aaaCucugaaugaacuuuuugg...................   8       1    seq
    ........................................................................aaaaucugaaugaacuuuuuggc..................   1       0    seq
    ........................................................................aaaaucugaaugaacuuuuuggU..................   14      1    seq
    .........................................................................aaaucugaaugaacuuuuu.....................   1       0    seq
    .........................................................................aaaucugaaugaacuuuuugg...................   1       0    seq
    ................................................................................aaugaacuuuuuggccaac..............   1       0    seq
    ................................................................................aaugaacuuuuuggccaacG.............   1       1    seq
```
